# Correlation between the hysteresis of the pressure–volume curve and the recruitment-to-inflation ratio in patients with coronavirus disease 2019

**DOI:** 10.1186/s13613-022-01081-x

**Published:** 2022-11-12

**Authors:** Ryuichi Nakayama, Naofumi Bunya, Shinshu Katayama, Yuya Goto, Yusuke Iwamoto, Kenshiro Wada, Keishi Ogura, Naoya Yama, Shintaro Takatsuka, Masumi Kishimoto, Kanako Takahashi, Ryuichiro Kakizaki, Keigo Sawamoto, Shuji Uemura, Keisuke Harada, Eichi Narimatsu

**Affiliations:** 1 Department of Emergency Medicine, Sapporo Medical University School of Medicine, South 1, West 16, 291, Minami 1-Jo Nishi 16-Chome, Chuo-Ku, Sapporo, Hokkaido 060-8556 Japan; 2 Division of Intensive Care, Department of Anesthesiology and Intensive Care Medicine, Jichi Medical University School of Medicine, Shimotsuke, Tochigi 329-0498 Japan; 3 Department of Intensive Care Medicine, School of Medicine, Sapporo Medical University, Sapporo, Hokkaido Japan; 4 Division of Radiology and Nuclear Medicine, Sapporo Medical University Hospital, Sapporo, Japan; 5 Department of Diagnostic Radiology, Sapporo Medical University School of Medicine, Sapporo, Japan; 6 Center for Medical Education, Sapporo Medical University, Sapporo, Japan; 7 Department of Clinical Engineering, Sapporo Medical University Hospital, Sapporo, Japan

**Keywords:** Respiratory distress syndrome, Mechanical ventilation, Coronavirus disease 2019

## Abstract

**Background:**

Since the response to lung recruitment varies greatly among patients receiving mechanical ventilation, lung recruitability should be assessed before recruitment maneuvers. The pressure–volume curve (PV curve) and recruitment-to-inflation ratio (R/I ratio) can be used bedside for evaluating lung recruitability and individualing positive end-expiratory pressure (PEEP). Lung tissue recruitment on computed tomography has been correlated with normalized maximal distance (NMD) of the quasi-static PV curve. NMD is the maximal distance between the inspiratory and expiratory limb of the PV curve normalized to the maximal volume. However, the relationship between the different parameters of hysteresis of the quasi-static PV curve and R/I ratio for recruitability is unknown.

**Methods:**

We analyzed the data of 33 patients with severe coronavirus disease 2019 (COVID-19) who received invasive mechanical ventilation. Respiratory waveform data were collected from the ventilator using proprietary acquisition software. We examined the relationship of the R/I ratio, quasi-static PV curve items such as NMD, and respiratory system compliance (*C*_rs_).

**Results:**

The median R/I ratio was 0.90 [interquartile range (IQR), 0.70–1.15] and median NMD was 41.0 [IQR, 37.1–44.1]. The NMD correlated significantly with the R/I ratio (rho = 0.74, *P* < 0.001). Sub-analysis showed that the NMD and R/I ratio did not correlate with *C*_rs_ at lower PEEP (− 0.057, *P* = 0.75; and rho = 0.15, *P* = 0.41, respectively). On the contrary, the ratio of *C*_rs_ at higher PEEP to *C*_rs_ at lower PEEP (*C*_rs_ ratio (higher/lower)) moderately correlated with NMD and R/I ratio (rho = 0.64, *P* < 0.001; and rho = 0.67, *P* < 0.001, respectively).

**Conclusions:**

NMD of the quasi-static PV curve and R/I ratio for recruitability assessment are highly correlated. In addition, NMD and R/I ratio correlated with the *C*_rs_ ratio (higher/lower). Therefore, NMD and R/I ratio could be potential indicators of recruitability that can be performed at the bedside.

**Supplementary Information:**

The online version contains supplementary material available at 10.1186/s13613-022-01081-x.

## Background

Patients with COVID-19 may require mechanical ventilation if they develop acute respiratory distress syndrome (ARDS) [1, 2]. In ARDS, the aerated lung area available for ventilation is reduced due to diffuse heterogeneous lung injury, alveolar collapse, and pulmonary edema (“baby lung”) [3, 4]. This means that the increased mechanical stress and strain on the reduced lung area increases the risk of ventilator-induced lung injury [5]. The open lung strategy for lung recruitment and individualized titration of PEEP to prevent alveolar collapse is expected to physiologically increase the well-aerated lung area, reduce atelectrauma; and improve lung compliance, intrapulmonary shunting, and oxygenation. However, the prognostic benefits in terms of ventilation-free days and mortality have not yet been demonstrated [6–9]. Therefore, there is ongoing controversy regarding the use of lung recruitment and the setting of optimal PEEP for patients with ARDS. This strategy may be useful in patients with moderate or severe ARDS with a PaO_2_/F_i_O_2_ ratio (P/F ratio) of  ≤ 200 mmHg [10–12].

As the response to the recruitment maneuver (RM) varies greatly among patients [13], one should assess lung recruitability before the RM to avoid overdistension of the open lung and negative cardiovascular effects [10, 11, 14]. Lung recruitability refers to the ability to re-aerate the non-aerated and poorly aerated lung tissue [14, 15]. The gold standard for the assessment of recruitability is the analysis of the lung area using computed tomography (CT) when PEEP changes [13, 16]. However, CT evaluation cannot be used routinely because of the considerable resources and risks involved in transporting patients on mechanical ventilation. The pressure–volume curve (PV curve) [15, 17] and recruitment-to-inflation ratio (R/I ratio) [14] can be used bedside for evaluating lung recruitability and individualing PEEP. Although there is a correlation between the R/I ratio and lung ultrasonographic findings [18], there is no study that validates the correlation between the R/I ratio and CT. Contrarily, the items of hysteresis generated by the low-flow quasi-static PV curve with a pressure setting of 5–45 cm H_2_O correlate with lung tissue recruitment on CT [17]. However, the relationship between the different parameters of hysteresis and R/I ratio is unclear. Therefore, the aim of this study was to evaluate the relationship between recruitability assessments based on the low-flow quasi-static PV curve and R/I ratio.

## Methods

### Study design

This was a retrospective, single-center cohort study of patients with COVID-19 who underwent invasive mechanical ventilatory management in the intensive care unit (ICU) of the Department of Emergency Medicine, Sapporo Medical University, Sapporo, Hokkaido, Japan, between January 1, 2021, and September 30, 2021. This study was conducted in accordance with the principles of the Declaration of Helsinki and was approved by the Ethics Committee of our institution (Approval number: 332-1138) on December 21, 2021. Since the study was retrospective in design, the need for informed consent was waived, and the patients and their families were guaranteed the opportunity to opt out.

### Patient population

All patients were diagnosed with COVID-19 by either polymerase chain reaction or quantitative antigen testing of nasal swabs and received mechanical ventilation using a Hamilton C6^®^ ventilator (Hamilton Medical AG, Rhäzüns, Switzerland). At our institution, based on the Japanese COVID-19 practice guidelines, ventilation is initiated for patients with COVID-19 who cannot maintain a SpO_2_ of 93% with noninvasive oxygen therapy [19]. As part of ventilatory management, the lung RM was introduced at our institution, along with prone position, neuromuscular blockade, and high PEEP for ventilated patients with COVID-19, in accordance with the guidelines of the American Thoracic Society, European Society of Intensive Care Medicine, and Society of Critical Care Medicine [20]. RM was performed after evaluation of the PV curve and R/I ratio as recruitability assessment.

The inclusion criteria were as follows: (1) 18 years of age or older, (2) COVID-19 patients who were ventilated using Hamilton C6^®^ during the study period, and (3) patients who underwent the quasi-static PV curve and the R/I ratio evaluated simultaneously with Datalogger 5.00 (Hamilton Medical AG, Rhäzüns, Switzerland). Patients who were younger than 18 years of age or whom respiratory data were not recorded by Datalogger 5.00 were excluded from the study.

The PV curve and the R/I ratio were assessed under assist/control ventilation with sedation and neuromuscular blockade (Additional file 1: Fig. S1).

### PV curve and airway closure

**Fig. 1:**
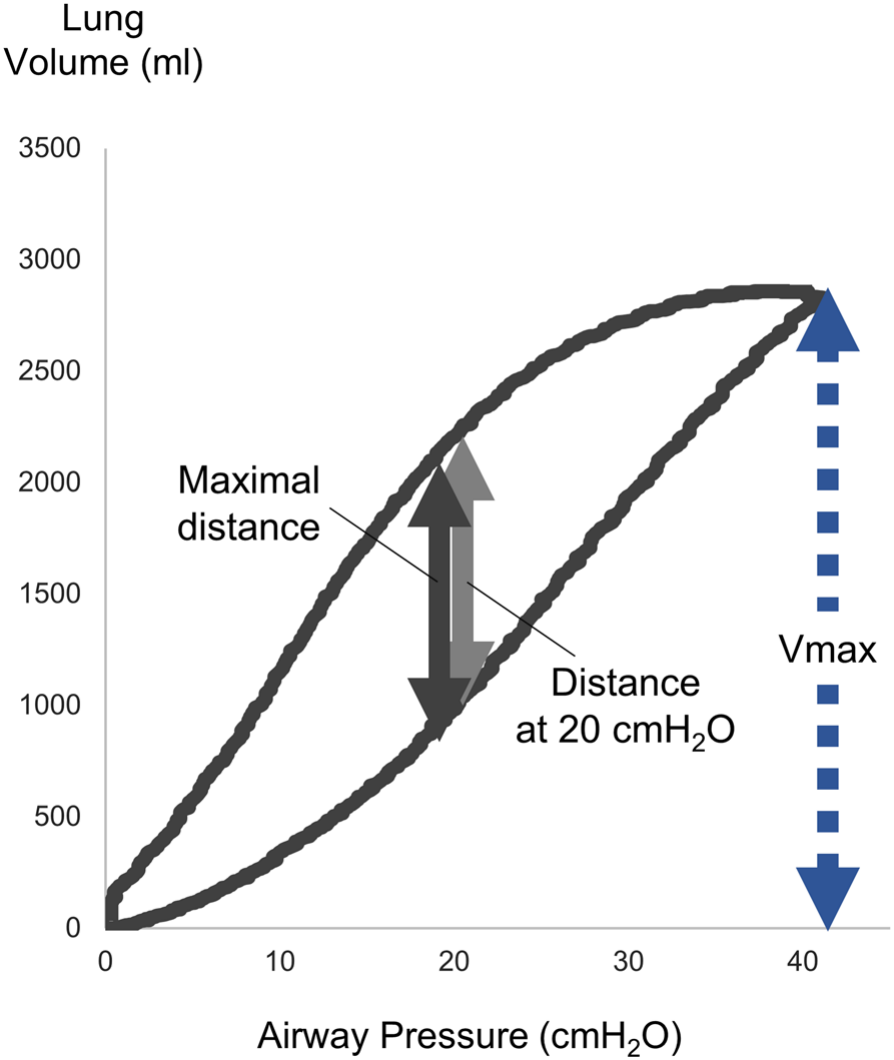
The respiratory system quasi-static pressure–volume curve and recruitment-to-inflation ratio. The respiratory system quasi-static pressure–volume curve was recorded using an automatic ventilator tool for low-flow inflation from 0 to 40 cm H_2_O and low-flow deflation from 40 to 0 cm H_2_O, with a constant pressure variation of 2 cm H_2_O/s. The evaluation parameters in the pressure–volume curve were as follows: volume difference between the inspiratory and expiratory limbs at 20 cm H_2_O (distance at 20 cm H_2_O); normalized distance at 20 cm H_2_O, which is the distance at 20 cm H_2_O weighted by the maximal volume (V_max_); maximal volume difference between the inspiratory and expiratory limbs at the same pressure (maximal distance); and normalized maximal distance, which is the maximal distance weighted by the V_max_

A quasi-static PV curve was drawn using a ventilator automatic tool (P/V tool; Hamilton Medical AG, Bonaduz, Switzerland) for low-flow inflation from 0–40 cm H_2_O and low-flow deflation from 40–0 cm H_2_O with a constant pressure variation of 2 cm H_2_O/s. The evaluation parameters of hysteresis in the PV curve included the volume difference between the inspiratory and expiratory limbs at 20 cm H_2_O (termed, distance at 20 cm H_2_O), which has been conventionally used [21]; the normalized distance at 20 cm H_2_O, which is the distance at 20 cm H_2_O weighted by the maximal volume (V_max_); the maximal volume difference between the inspiratory and expiratory limbs at the same pressure (maximal distance); and the normalized maximal distance (NMD), which is the maximal distance weighted by the V_max_ (Fig. 1) [17].

Airway closure, which can sometimes occur in ARDS, is a phenomenon in which the airway and alveoli cannot communicate with each other without the application of a certain amount of pressure, termed the airway opening pressure (AOP) [22]. AOP was identified as the lower inflection point in the PV curve with compliance as low as 1.5–2.5 mL/cm H_2_O above 5 cm H_2_O [23, 24].

### R/I ratio

By using the single-breath method to reduce the PEEP from higher to lower pressure (typically from 15 or 18 cm H_2_O to 5 or 8 cm H_2_O) in a single breath and to calculate respiratory system compliance (*C*_rs_) at lower PEEP, we recorded the tidal change in end-expiratory lung volume between the two PEEP levels (measured ΔEELV) and estimated the predicted ∆EELV in the absence of recruitment effect by PEEP. The recruited volume (ΔV_rec_) was calculated by subtracting the predicted from the measured ΔEELV. The pressure contributing to recruitment (∆P_rec_) was defined as the difference between the higher and lower PEEP, or as the difference between higher PEEP and AOP, if it existed. Further, compliance of the recruited lung (*C*_rec_) was the value obtained by dividing ∆V_rec_ by ∆P_rec_. The R/I ratio can be calculated as a ratio of *C*_rec_ to *C*_rs_ at lower PEEP (5 or 8 cm H_2_O, or above AOP), which is considered as a surrogate for the compliance of the ‘baby lung’. The higher the R/I ratio, the greater the lung recruitability [14].

### Procedure of recruitability assessment

The patients were ventilated for at least 2 min with a high PEEP, a single-breath maneuver was performed to reduce PEEP (from 15 or 18 to 5 or 8 cm H_2_O), and the plateau pressure was measured at least 2 min later. This was adopted on the basis that the change in end-expiratory volume stabilizes at 2 min with increasing or decreasing PEEP [25]. The respiratory rate was reduced to 8–10 breaths/min before this procedure to limit auto-PEEP. Recruitability assessment was evaluated in the supine flat position.

### Data collection and measurements

We retrospectively collected patient data from the hospital’s electronic medical records documented at the time of admission and assessment of recruitability. The baseline patient characteristics obtained at admission were age, sex, height, body weight, body mass index (BMI), pre-existing conditions, and Acute Physiology and Chronic Health Evaluation II score. The following respiratory parameters were obtained at the time of the recruitability assessment: P/F ratio, duration of ventilation, the Sequential Organ Failure Assessment score, tidal volume divided by predicted body weight, PEEP, plateau pressure, and respiratory system compliance.

Airway pressure and flow were measured using the proximal pneumotachograph of the ventilator (single-use flow sensor, PN 281,637; Hamilton Medical AG, linear between − 260 and + 260 L/min with a ± 10% or ± 20 mL/s error of measure) placed between the endotracheal tube and Y-piece. Respiratory waveform data were collected from the ventilator using proprietary acquisition software (Datalogger 5.00, Hamilton Medical AG, Rhäzüns, Switzerland).

### Data analysis

Data are expressed as median and interquartile range (IQR). The correlation between different variables was assessed using Spearman’s rank correlation coefficient with 95% confidence interval (CI). Analyses were performed using R software version 4.1.2 (The R Foundation for Statistical Computing, Vienna, Austria).

The primary outcome was the correlation between the items of the different recruitability indicators (R/I ratio vs. NMD, maximal distance, distance at 20 cm H_2_O, and normalized distance at 20 cm H_2_O). Furthermore, to evaluate the relationship between recruitability and *C*_rs_, which correlates with the amount of normally aerated tissue [4], the correlation between the items of compliance (*C*_rs_ at higher and lower PEEP, and the ratio of *C*_rs_ at higher PEEP [15 or 18 cm H_2_O] to *C*_rs_ at lower PEEP [5 or 8 cm H_2_O] [termed, *C*_rs_ ratio (higher/lower)]) and the main items of recruitability (R/I ratio, NMD) were also examined.

## Results

### Enrollment and baseline characteristics

**Fig. 2:**
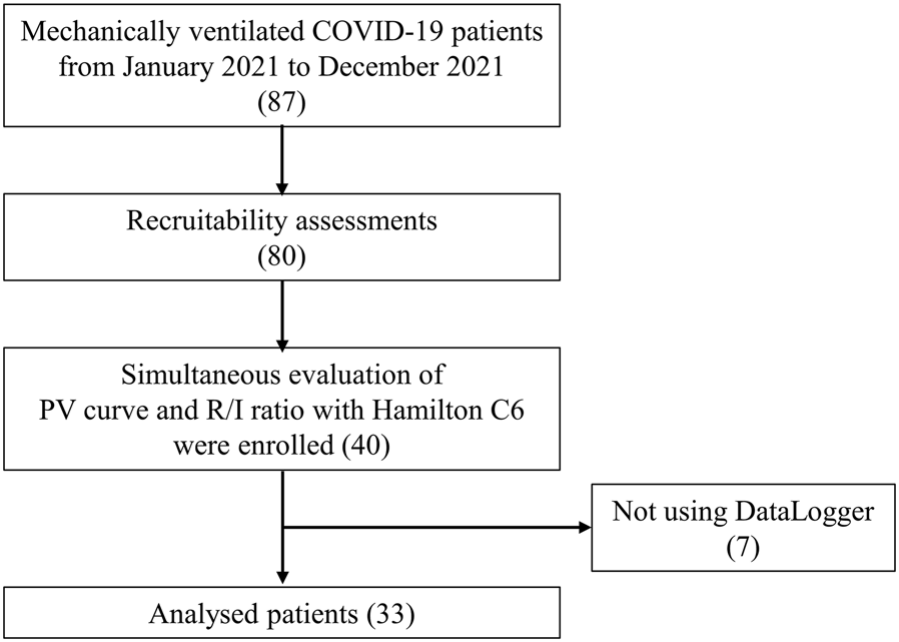
Flowchart of the study patients

During the study period, 87 Japanese patients with COVID-19 were mechanically ventilated, and recruitability assessments were performed on 80 patients. As illustrated in the flowchart (Fig. 2), 40 patients who underwent the quasi-static PV curve and the R/I ratio were enrolled. Seven patients were excluded from the analysis since the data were not collected by DataLogger. The baseline patient characteristics are presented in Table 1.

**Table 1:** Patient characteristics, respiratory mechanics, and recruitability assessment

**Parameter**	*n* = 33
Age, years	57 [50–64]
Sex, M, *n* (%)	21 (63.6)
Height, cm	168.0 [156.0–172.0]
Body weight, kg	74.3 [62.0–85.0]
Body mass index, kg/m^2^	27.5 [23.8–29.0]
Pre-existing conditions, *n* (%)	
COPD	12 (36.4)
Hypertension	12 (36.4)
Diabetes	26 (78.8)
Receiving high-flow nasal cannula before invasive mechanical ventilation, *n* (%)	12 (36.4)
Receiving prone position after invasive mechanical ventilation, *n* (%)	32 (97.0)
Interval between onset and admission	10 [6–11]
Severity of ARDS at admission, *n* (%)	
Mild	5 (15.2)
Moderate	18 (54.6)
Severe	8 (24.2)
KL-6 at admission	571 [318–841]
APACHE II at admission	13 [8–15]
Interval between start of mechanical ventilation and recruitability assessment, days	0 [0–1]
P_a_O_2_/F_i_O_2_ at recruitability assessment, mmHg	116.0 [90.0–183.0]
SOFA score at recruitability assessment	3 [2–7]
Respiratory data at recruitability assessment	
TV, mL/kg (PBW)	6.2 [5.8–7.4]
PEEP, cm H_2_O	15 [14, 15]
P_plat_, cm H_2_O	25 [23–29]
*C*_rs_, mL/cm H_2_O	36.6 [30.1–44.4]
Recruitability assessment	
Airway opening pressure > 5 cm H_2_O, *n* (%)	7 (21.2)
Higher PEEP for R/I ratio, cm H_2_O	15 [15–15]
Set tidal volume for R/I ratio, mL	400 [400–400]
VT exhaled at higher PEEP for R/I ratio, mL	390 [380–400]
Lower PEEP for R/I ratio, cm H_2_O	5 [5–5]
VT exhaled from higher to lower PEEP, mL	1145 [959–1280]
Plateau pressure at lower PEEP for R/I ratio, cm H_2_O	15 [14–18]
∆V_rec_, mL	334.3 [260.0–451.4]
R/I ratio	0.90 [0.70–1.15]
Distance at 20 cm H_2_O, mL	810 [645–970]
Maximal distance, mL	845 [670–995]
Airway pressure at maximal distance, cm H_2_O	19.5 [17.9–20.4]
Maximal volume (V_max_), mL	2065 [1710–2625]
Normalized distance at 20 cm H_2_O, %	39.9 [35.8–42.6]
Normalized maximal distance, %	41.0 [37.1–44.1]

Data are presented as median [IQR] *n* (%), where *n* is the total number of patients with COVID-19 in the relevant data minus the missing values

*COVID-19* coronavirus disease 2019, *COPD* chronic obstructive pulmonary disease, *ARDS* acute respiratory distress syndrome, *KL-6* Krebs von den Lungen-6, *APACHE II* Acute Physiologic Assessment and Chronic Health Evaluation II, *SOFA* Sequential Organ Failure Assessment, *TV* tidal volume, *PEEP*, positive end-expiratory pressure, _*Pplat*_ plateau pressure, *C*_*rs*_ respiratory system compliance, *R/I ratio* recruitment-to-inflation ratio

### Recruitability assessment of NMD and R/I ratio

**Fig. 3:**
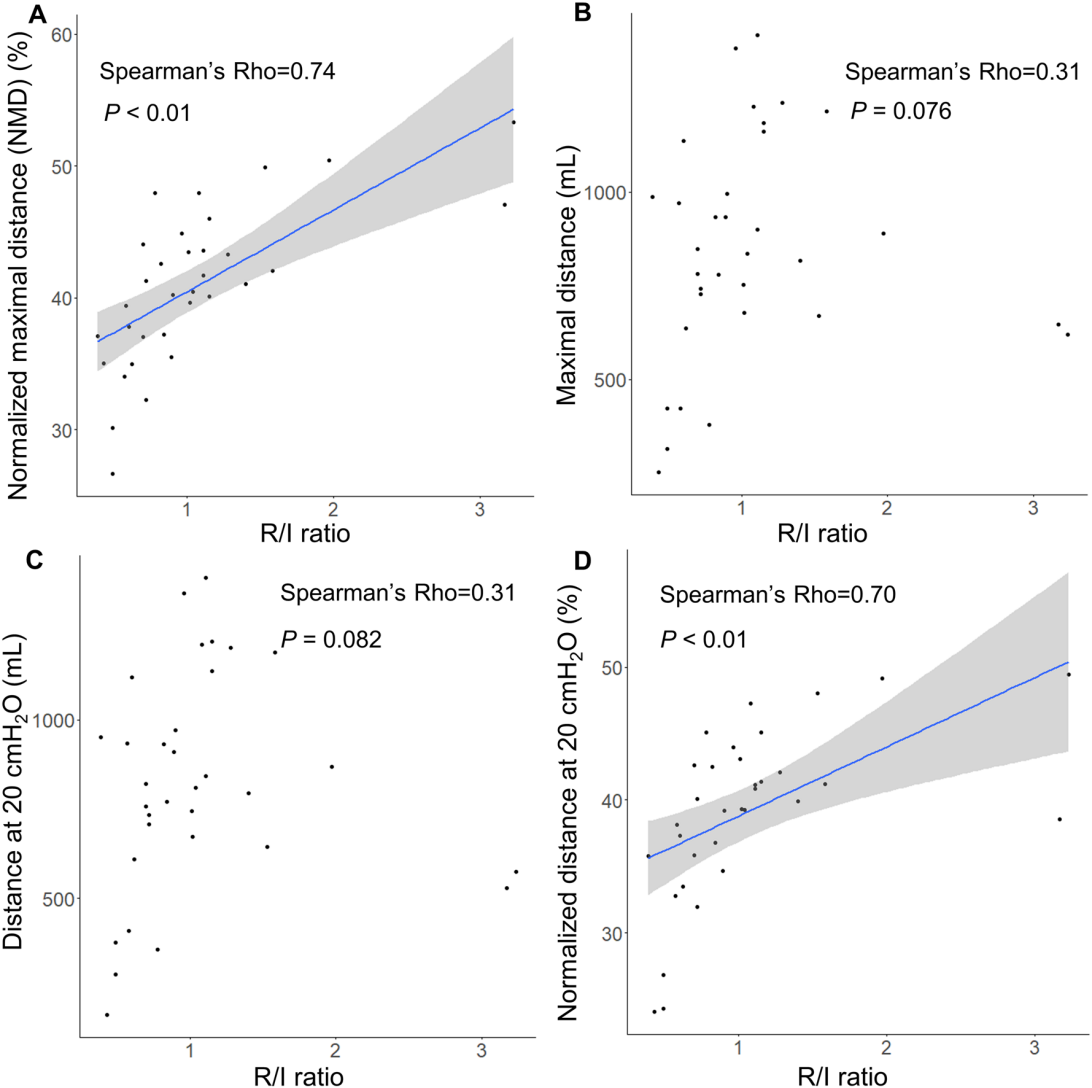
Correlation between the items of the different recruitability indicators. **A** The correlation between the items of the different recruitability indicators showing a significant correlation between the recruitment-to-inflation ratio (R/I ratio) and the normalized maximal distance (rho = 0.74, *P* < 0.001). **B** The relationship between the maximal distance and the R/I ratio (rho = 0.31, *P* = 0.076,), and **C** between distance at 20 cm H_2_O and the R/I ratio (rho = 0.31, *P* = 0.082) were not correlated. **D** Moderate correlation was observed between normalized distance at 20 cm H_2_O and the R/I ratio (rho = 0.70, *P* < 0.001)

The NMD of the PV curve correlated significantly with the R/I ratio (rho = 0.74 [95% CI 0.52 to 0.87], *P* < 0.001, Fig. 3A). The relationship between maximal distance and the R/I ratio (rho = 0.31 [95% CI − 0.054 to 0.64], *P* = 0.076, Fig. 3B) and between distance at 20 cm H_2_O and the R/I ratio (rho = 0.31 [95% CI − 0.086 to 0.62], *P* = 0.082, Fig. 3C) were not correlated. Moderate correlation was observed between normalized distance at 20 cm H_2_O and the R/I ratio (rho = 0.70 [95% CI 0.44 to 0.86] *P* < 0.001, Fig. 3D). Analysis of the patients whose airway pressure at maximal distance in the PV curve were below 20 cm H_2_O showed a strong correlation between the NMD and R/I ratio (*n* = 23, rho = 0.80 [95% CI 0.58 to 0.91], *P* < 0.001, Additional file 2: Fig. S2). Additional file 4: Table S4 shows the correlation matrix of recruitability items.

### Relationship between compliance and recruitability items

**Fig. 4:**
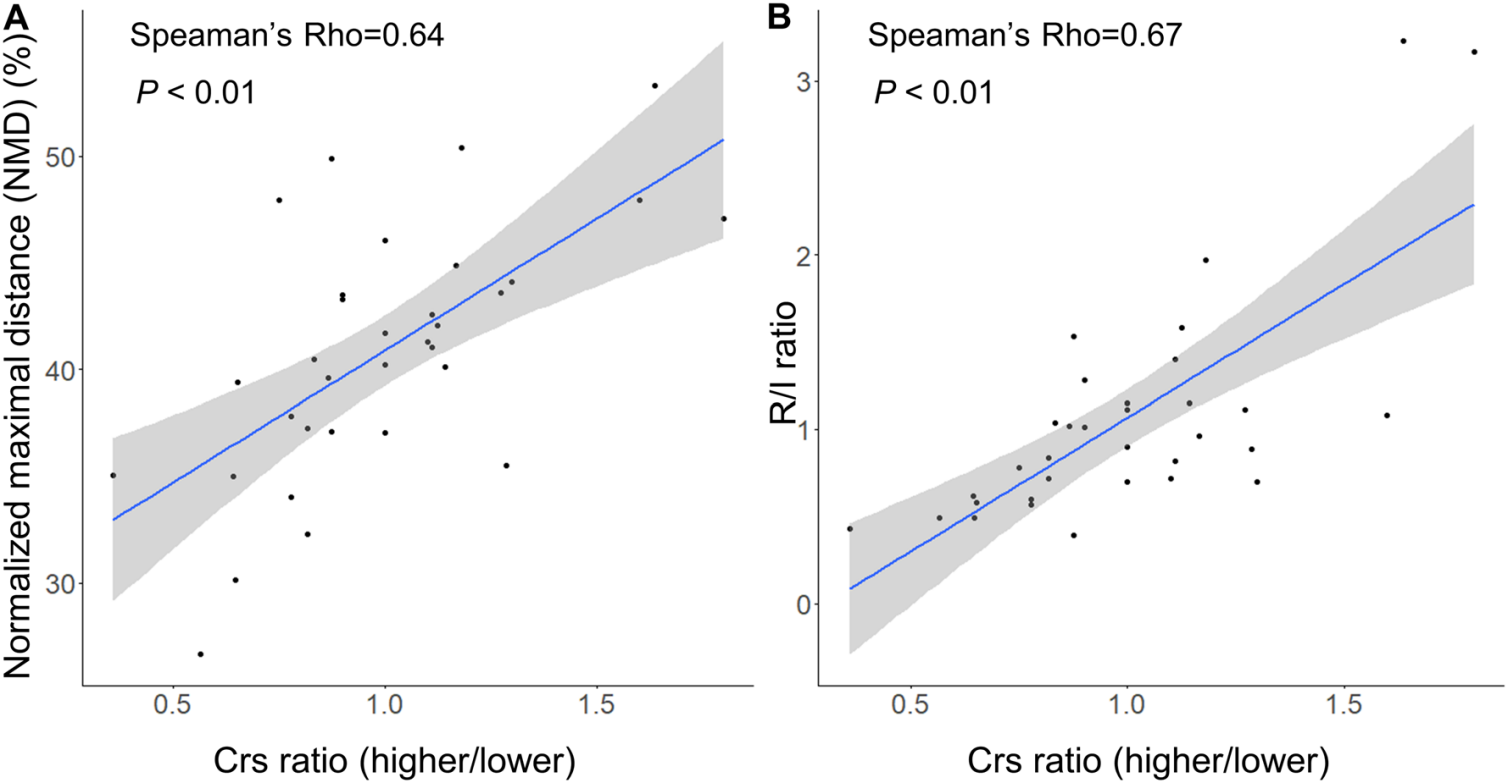
Correlation between compliance and recruitability items. **A** The ratio of respiratory system compliance (*C*_rs_) at higher PEEP (15 or 18 cm H_2_O) to *C*_rs_ at lower PEEP (5 or 8 cm H_2_O) (termed, *C*_rs_ ratio (higher/lower)) moderately correlated with NMD (rho = 0.64, *P* < 0.001) and **B** R/I ratio (rho = 0.67, *P* < 0.001)

*C*_rs_ at higher PEEP in the procedure of R/I ratio did not correlate with NMD (rho = 0.14, *P* = 0.43) and R/I ratio (rho = 0.27, *P* = 0.13) (Additional file 3: Fig. S3). On the contrary, *C*_rs_ ratio (higher/lower) moderately correlated with NMD and R/I ratio (respectively, rho = 0.64, *P* < 0.001 and rho = 0.67, *P* < 0.001) (Fig. 4).

## Discussion

In this study, we found a significantly higher correlation between NMD and R/I ratio than between distance at 20 cm H_2_O and R/I ratio. In addition, NMD and R/I ratio did not correlate with compliance of respiratory system but correlated with *C*_rs_ ratio (higher/lower).

This study indicated a correlation between the R/I ratio and the NMD of hysteresis. Though the PV curve is available in only a limited number of ventilators (e.g., Hamilton C6^®^) [26], the R/I ratio is available in any ventilator; however, it has not been validated with reference to CT and hysteresis which correlated with lung recruitment on CT scan [17]. As shown in Additional file 4: Table S4, apart from normalized distance at 20 cm H_2_O which is similar in nature to NMD, the R/I ratio correlates best with NMD among recruitability items, such as distance at 20 cm H_2_O. Unfortunately, the correlation between NMD and R/I ratio was not perfect. One possible explanation is that the maximal distance and *C*_rec_ are similar yet different phenomena. This is because the maximal distance is the maximum volume difference between the inspiratory and expiratory curves of the PV curve, while *C*_rec_ is the volume recruited by PEEP. Furthermore, since the correlation coefficient between NMD and R/I ratio was higher in the patients whose airway pressure at maximal distance in the PV curve were below 20 cm H_2_O (Additional file 2: Fig. S2), the R/I ratio could indicate a pressure volume relationship at 15 cm H_2_O or 18 cm H_2_O and may be unsuitable for evaluation at higher pressures. Hence, the PV curve represented by NMD is considered more informative than the R/I ratio. However, the R/I ratio may be preferable when pressure limitation is desired, such as in cases of barotrauma.

The R/I ratio and the NMD did not correlate with *C*_rs_ at lower PEEP and *C*_rs_ at higher PEEP. Gattinoni et al. proposed that for COVID-19 pneumonia, Type L with low recruitability indicated high compliance (low elastance) and Type H with high recruitability indicated low compliance (high elastance) [27]; yet no such correlation was derived from our study. In this study, there were cases with low recruitability and low compliance (less than 30 mL/cm H_2_O), and cases with high recruitability and normal compliance. This discussion is indicative of the heterogeneous nature of ARDS and would require further validation. The authors believe that compliance alone is inconsistent with the evaluation of recruitability, and therefore, R/I and NMD should be used to actively evaluate recruitability.

*C*_rs_ ratio (higher/lower) indicates the degree of improvement in *C*_rs_ at a higher PEEP (such as 15 cm H_2_O) compared to *C*_rs_ at lower PEEP (such as 5 cm H_2_O). The correlation of NMD and R/I ratio with the *C*_rs_ ratio (higher/lower) is consistent with the concept of recruitability in terms of improved compliance. A previous study has also shown better compliance with higher PEEP than a lower PEEP in the group with high recruitability on CT [13]. Hence, NMD and R/I ratio could be potential indicators of recruitability.

### Limitations

This research has several limitations. First, this is a single-center retrospective observational study. The sample size was small and not all patients were evaluated. Second, to improve the accuracy of the data, seven patients were excluded from the analysis because their information was not collected by Datalogger. Third, this study set the time for measuring the plateau pressure in the single-breath method of R/I ratio to at least 2 min based on a previous study [25]. However, other studies have shown that lung volume equilibrium may require more time [28]. This point should be verified in a future prospective study. Fourth, due to infection control issues, we lacked CT data with varying PEEP as a reference for assessing lung recruitability. In future studies, it would be worthwhile to compare the R/I ratio and PV curve using CT as a reference. Fifth, contrary to the report by Chen et al. (Servo-I, GETINGE) [14], we used low-flow, constant-pressure inflation and a proximal pneumotachograph (PN 281637; Hamilton Medical AG). We observed that airway closure does indeed occur even with a pressure-constant, quasi-static PV curve. Future studies are needed to determine if the detected AOPs are equivalent to flow-constant and pressure-constant quasi-static PV curves.

## Conclusions

NMD of the quasi-static PV curve and R/I ratio for recruitability assessment are highly correlated. In addition, NMD and R/I ratio correlated with the *C*_rs_ ratio (higher/lower). Therefore, NMD and R/I ratio could be potential indicators of recruitability that can be performed at the bedside.

## Supplementary Information

**Additional file 1: Figure S1.** Combined procedure of recruitability assessment including the recruitment-to-inflation ratio and pressure–volume curve.

**Additional file 2: Figure S2.** Correlation between the NMD and the R/I ratio in the patients within 20 cm H_2_O of airway pressure at maximum distance in the PV curve. Analysis in the patients within 20 cm H_2_O of airway pressure at maximum distance in the PV curve showed a strong correlation between the NMD and the R/I ratio (*n* = 23, rho = 0.80 [95% CI 0.58 to 0.91], *P* < 0.001).

**Additional file 3: Figure S3.** Scatter diagrams between recruitability assessment and respiratory system compliance (*C*_rs_). The relationship between the NMD and *C*_rs_ at higher PEEP (A), and between the R/I ratio and *C*_rs_ at higher PEEP (B) were all both correlated.

**Additional file 4: Table S4.** Matrix of Spearman’s correlation coefficients of recruitability items.

## Data Availability

The datasets used and/or analyzed during the current study are available from the corresponding author upon reasonable request.

## Abbreviations

∆P_rec_: Pressure contributing to recruitment
AOP: Airway opening pressure
ARDS: Acute respiratory distress syndrome
BMI: Body mass index
CI: Confidence interval
COVID-19: Coronavirus disease 2019
C_rec_: Compliance of the recruited lung
C_rs_: Respiratory system compliance
CT: Computed tomography
ICU: Intensive care unit
IQR: Interquartile range
NMD: Normalized maximal distance
P/F ratio: PaO_2_/F_i_O_2_ ratio
PEEP: Positive end-expiratory pressure
P_plat_: Plateau pressure
PV curve: Pressure–volume curve
R/I ratio: Recruitment-to-inflation ratio
RM: Recruitment maneuver
Vmax: Maximal volume
ZEEP: Zero positive end-expiratory pressure
ΔEELV: Tidal change in end-expiratory lung volume
ΔV_rec_: Recruited volume
