# Supplementary figures and images for: Correlation between the hysteresis of the pressure–volume curve and the recruitment-to-inflation ratio in patients with coronavirus disease 2019
Source: Ann Intensive Care. 2022 Nov 12;12:106. doi: 10.1186/s13613-022-01081-x (PMC9652597; doi:10.1186/s13613-022-01081-x)

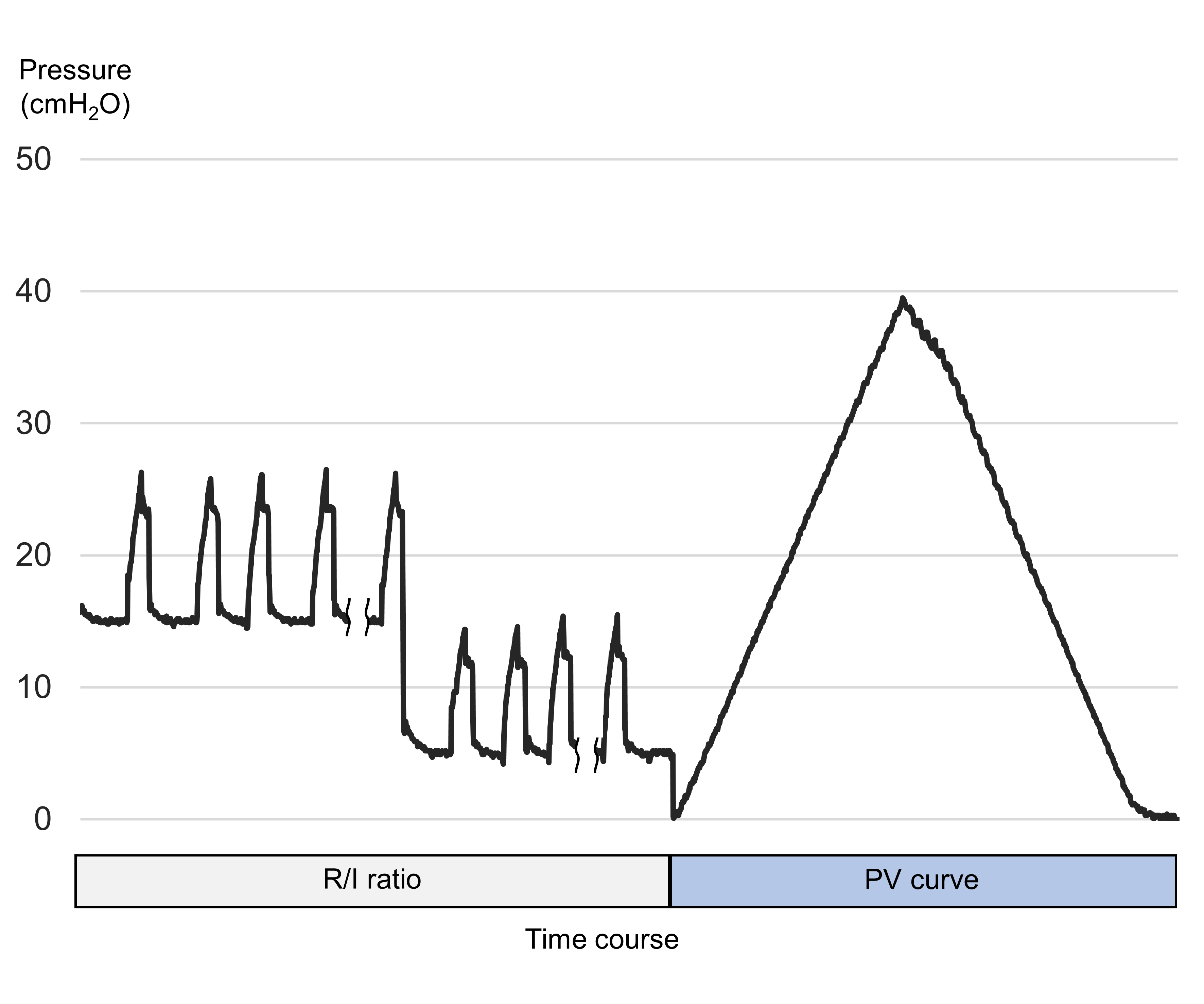

Supplement: Supplementary file 1 — Additional file 1: Figure S1. Combined procedure of recruitability assessment including the recruitment-to-inflation ratio and pressure–volume curve. [file 13613_2022_1081_MOESM1_ESM.tif]

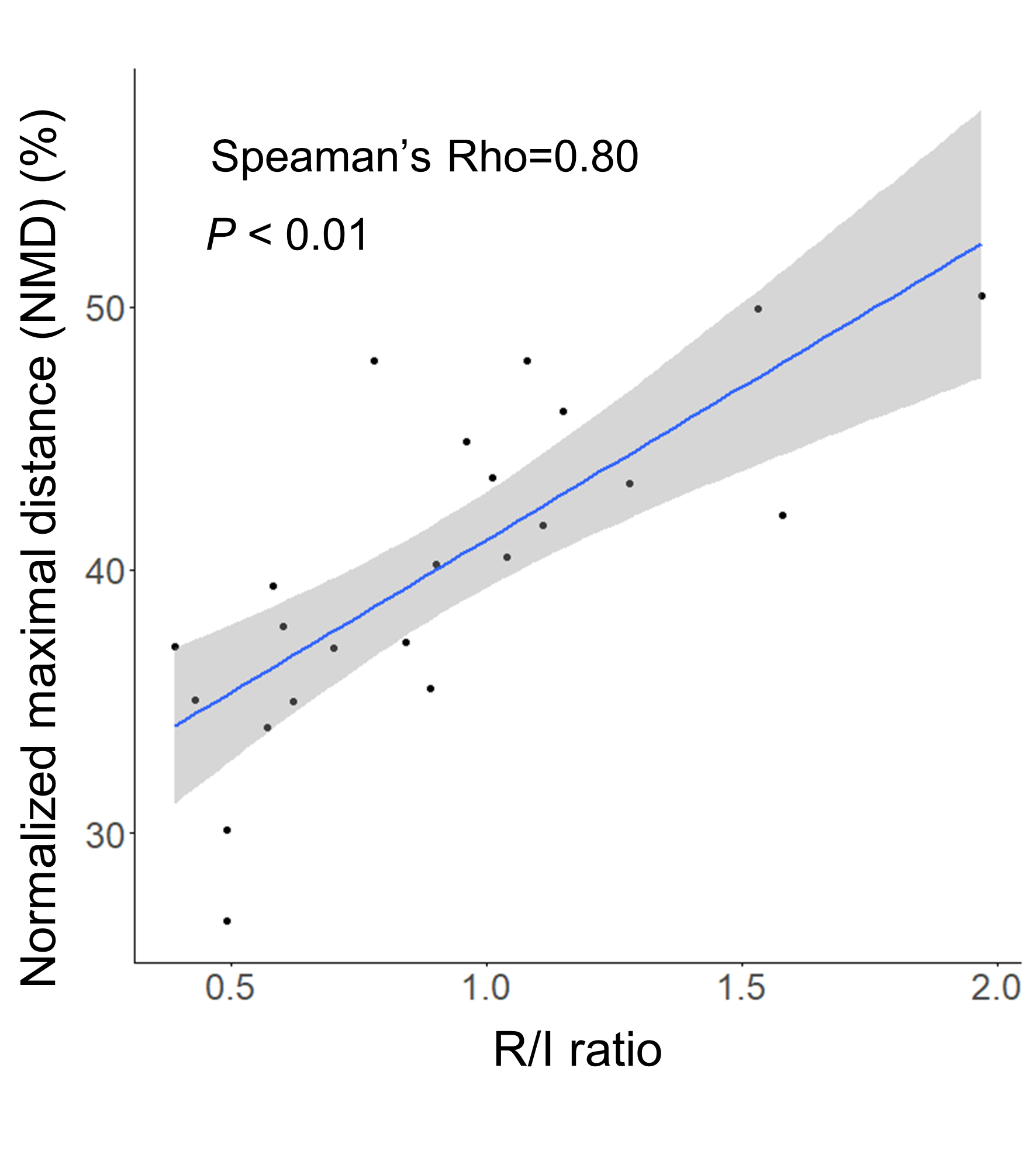

Supplement: Supplementary file 2 — Additional file 2: Figure S2. Correlation between the NMD and the R/I ratio in the patients within 20 cm H2O of airway pressure at maximum distance in the PV curve. Analysis in the patients within 20 cm H2O of airway pressure at maximum distance in the PV curve showed a strong correlation between the NMD and the R/I ratio (n = 23, rho = 0.80 [95% CI 0.58 to 0.91], P < 0.001). [file 13613_2022_1081_MOESM2_ESM.tif]

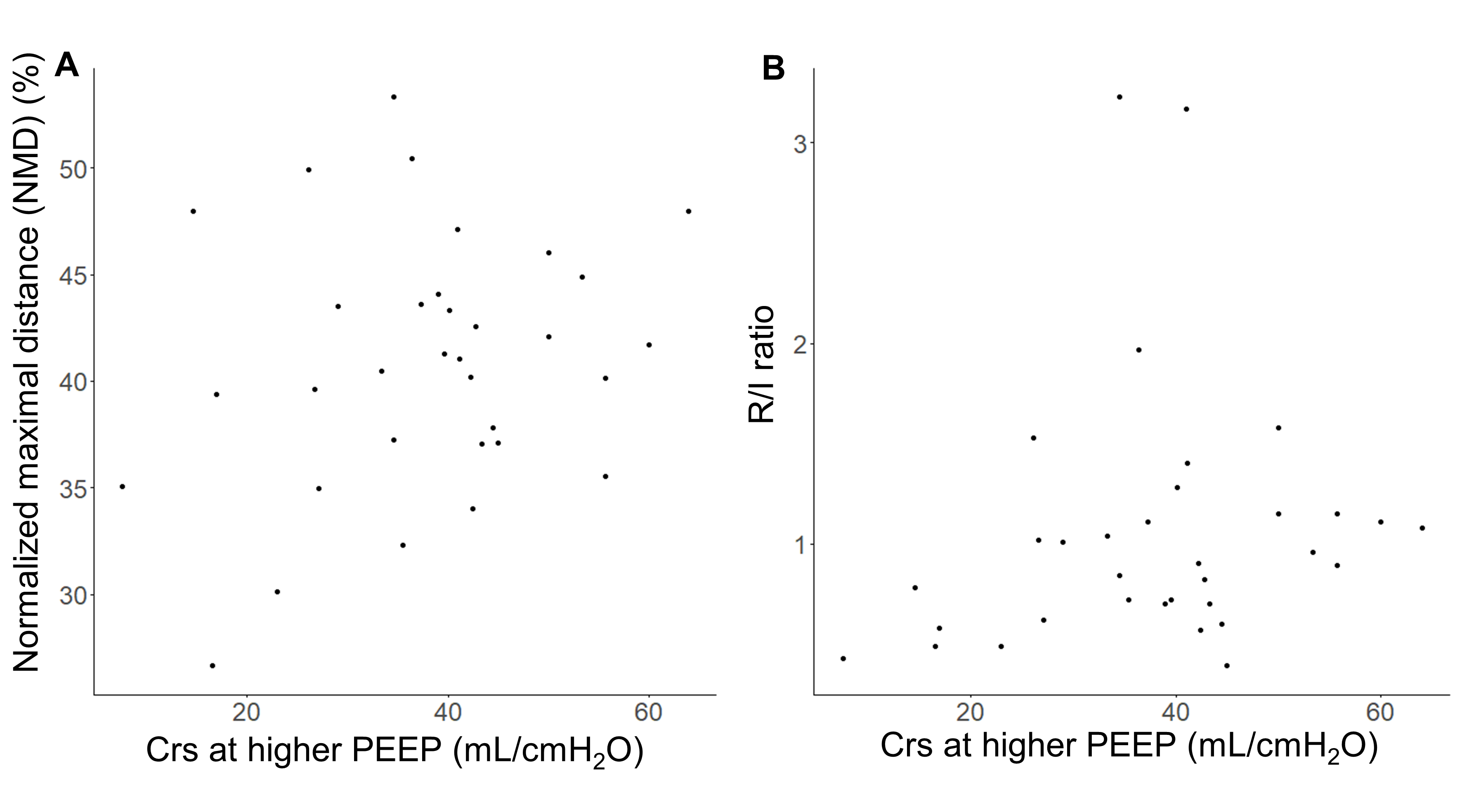

Supplement: Supplementary file 3 — Additional file 3: Figure S3. Scatter diagrams between recruitability assessment and respiratory system compliance (Crs). The relationship between the NMD and Crs at higher PEEP (A), and between the R/I ratio and Crs at higher PEEP (B) were all both correlated. [file 13613_2022_1081_MOESM3_ESM.tif]
