# Supplementary material for: Correlation between the hysteresis of the pressure–volume curve and the recruitment-to-inflation ratio in patients with coronavirus disease 2019
Source: Ann Intensive Care. 2022 Nov 12;12:106. doi: 10.1186/s13613-022-01081-x (PMC9652597; doi:10.1186/s13613-022-01081-x)
Supplement: Supplementary file 4 — Additional file 4: Table S4. Matrix of Spearman’s correlation coefficients of recruitability items. [file 13613_2022_1081_MOESM4_ESM.docx]

| **Additional file Table.**  **Matrix of Spearman's correlation coefficients of recruitability items** | | | | | | | | |
| --- | --- | --- | --- | --- | --- | --- | --- | --- |
|  | **R/I ratio** | **C_rs_ at lower PEEP** | **ΔV_rec_** | **NMD** | **Normalized distance at 20 cmH_2_O** | **Maximal distance** | **Distance at 20 cmH_2_O** | **V_max_** |
| **R/I ratio** |  |  |  |  |  |  |  |  |
| **C_rs_ at lower PEEP** | 0.15 |  |  |  |  |  |  |  |
| **ΔV_rec_** | 0.64^**^ | 0.61^**^ |  |  |  |  |  |  |
| **NMD** | 0.74^**^ | −0.057 | 0.32 |  |  |  |  |  |
| **Normalized distance at 20 cmH_2_O** | 0.70^**^ | 0.012 | 0.44^*^ | 0.94^**^ |  |  |  |  |
| **Maximal distance** | 0.31 | 0.74^**^ | 0.82^**^ | 0.20 | 0.30 |  |  |  |
| **Distance at 20 cmH_2_O** | 0.31 | 0.75^**^ | 0.83^**^ | 0.19 | 0.31 | 1.00^**^ |  |  |
| **V_max_** | 0.11 | 0.81^**^ | 0.73^**^ | −0.092 | 0.027 | 0.93^**^ | 0.93^**^ |  |

* *P* < 0.05, ** *P* < 0.01

V_max_, maximal volume; C_rs_, respiratory system compliance; ΔV_rec_, recruited volume; R/I ratio, recruitment-to-inflation ratio; NMD, normalized maximal distance
